# Supplementary figures and images for: Developing a simple, cost-effective proof-of-concept vaccine candidate against EHEC for cattle
Source: PLoS One. 2026 Jan 27;21(1):e0341075. doi: 10.1371/journal.pone.0341075 (PMC12843572; doi:10.1371/journal.pone.0341075)

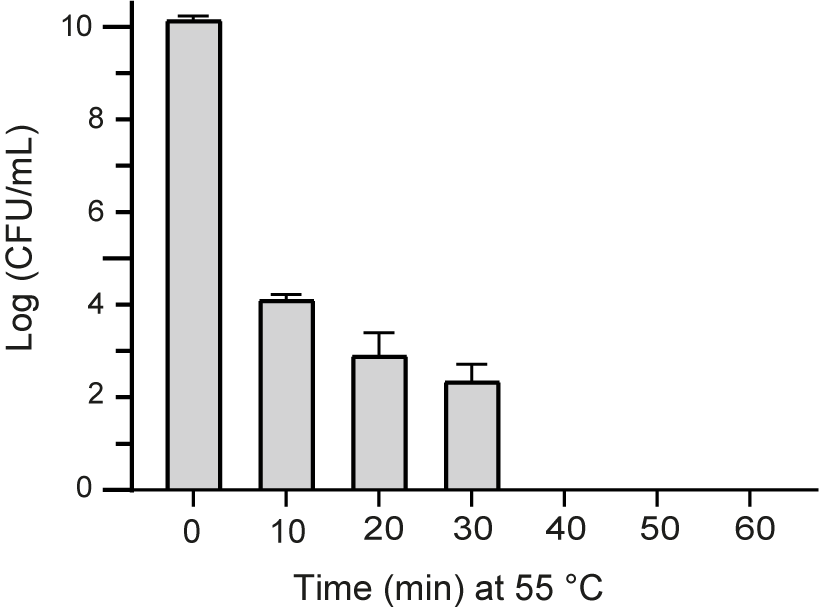

Supplement: S1 Fig — Bacterial pellets were resuspended in TE buffer and incubated at 55 °C. Every 10 min, aliquots were plated for CFU determination. (TIF) [file pone.0341075.s001.tif]

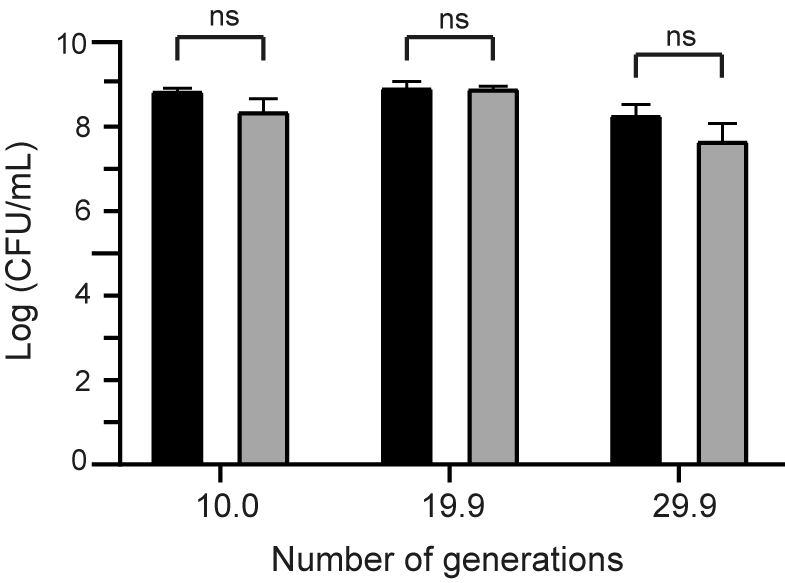

Supplement: S2 Fig — After successive cultivation in antibiotic-free media, aliquots were plated on both LB with (black bars) and without (grey bars) kanamycin for CFU counting. Error bars correspond to standard deviations from replicates, data were analyzed by Student´s t-tests. ns: not significant. (TIF) [file pone.0341075.s002.tif]

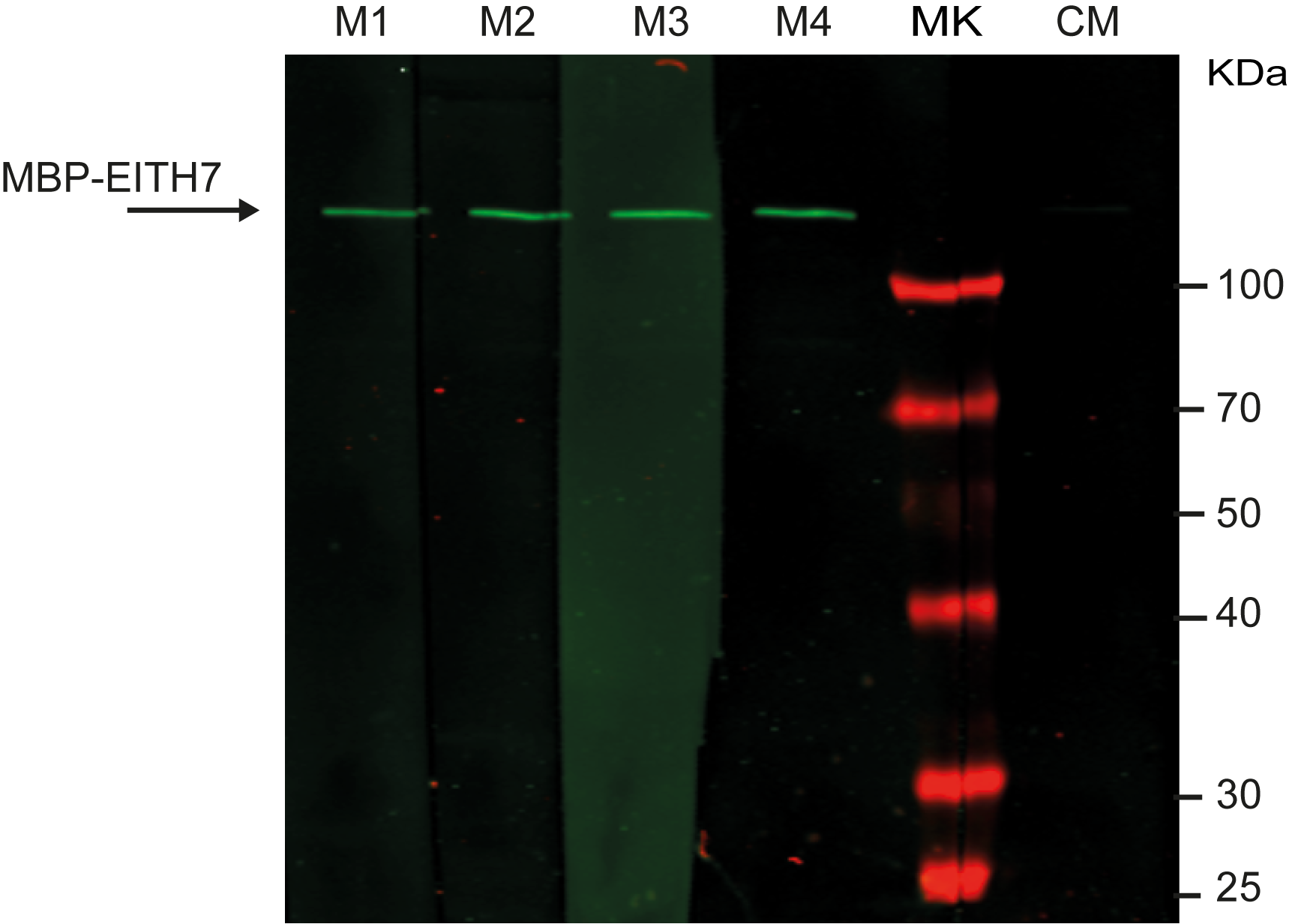

Supplement: S3 Fig — To test antibody specificity, purified MBP-EITH7 was run in SDS-PAGE and revealed with sera of 4 mice at 4 weeks post-immunization (M1 to M4) and a control mouse (CM) as primary antibodies. (TIF) [file pone.0341075.s003.tif]

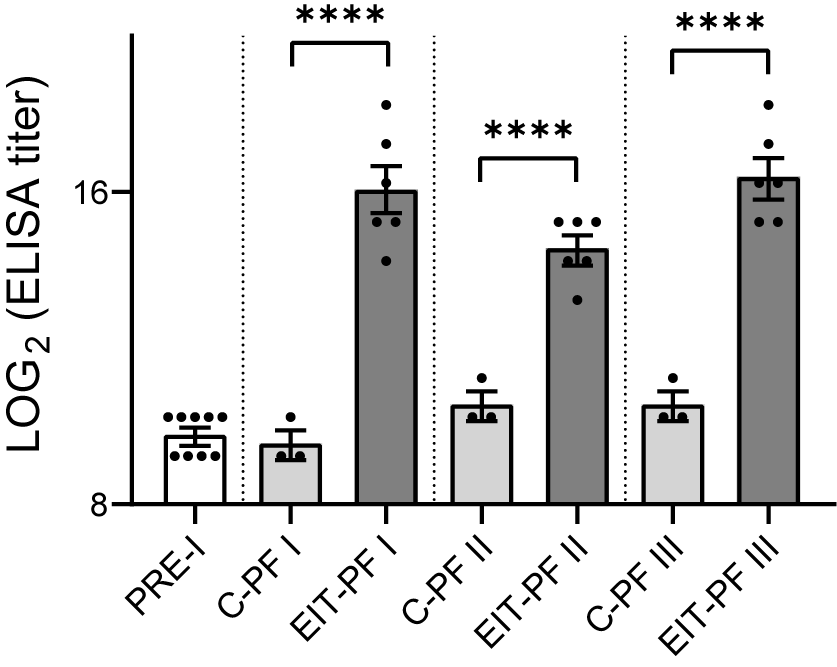

Supplement: S4 Fig — Endpoint titers were determined for sera from control and EIT-immunized steers at the different post immunization days. ELISA endpoint titers are expressed as the base 2 logarithm of the reciprocal of the maximum dilution that resulted in an Abs 450nm value above the cut-off. The cut-off was defined as the average + 3SD of the levels measured for pre-immune sera. Each dot represents individual immunized steers (EIT-PF), or the control steer (C-PF) determined in triplicate, and the averages (±SEM) are shown for all data. PRE-I corresponds to pre-immune sera; C-PF I, II, III and EIT-PF I, II and III correspond to sera from steers immunized with control periplasmic fraction or EIT periplasmic fraction at week 2 (I dose), 4 (II doses) and 6 (III doses), respectively. Data were analyzed by One-way ANOVA and Sidak’s post hoc contrasts; asterisks indicate a p-value < 0.0001 (****). (TIF) [file pone.0341075.s004.tif]

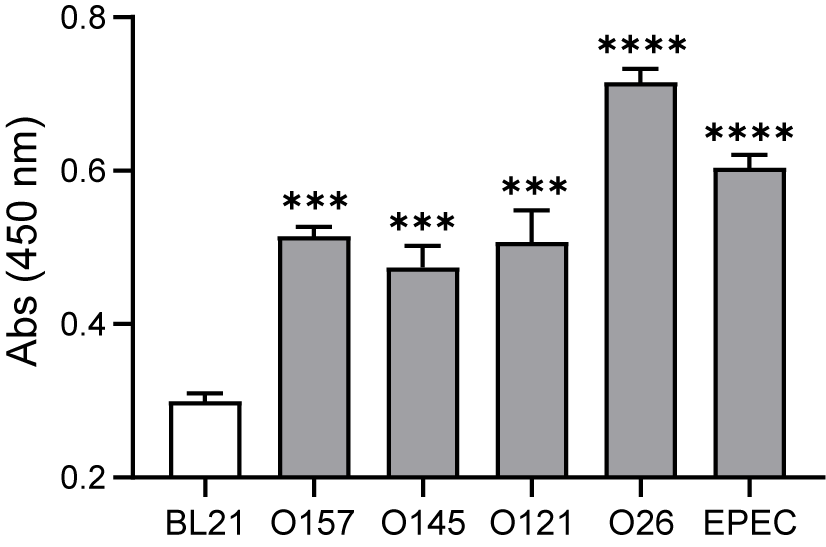

Supplement: S5 Fig — Cross-reactivity of IgG antibodies was tested by coating ELISA plates with lysates of individual pathogen serotypes. Sera from one-dose EIT-PF immunized steers (week 2 p.i.) were pooled and assayed in triplicate, and the averages (±SEM) are shown. Data were analyzed by One-way ANOVA and Dunnett contrasts comparing to BL21 strain. Asterisks indicate a p-value < 0.001 (***) or < 0.0001 (****). (TIF) [file pone.0341075.s005.tif]

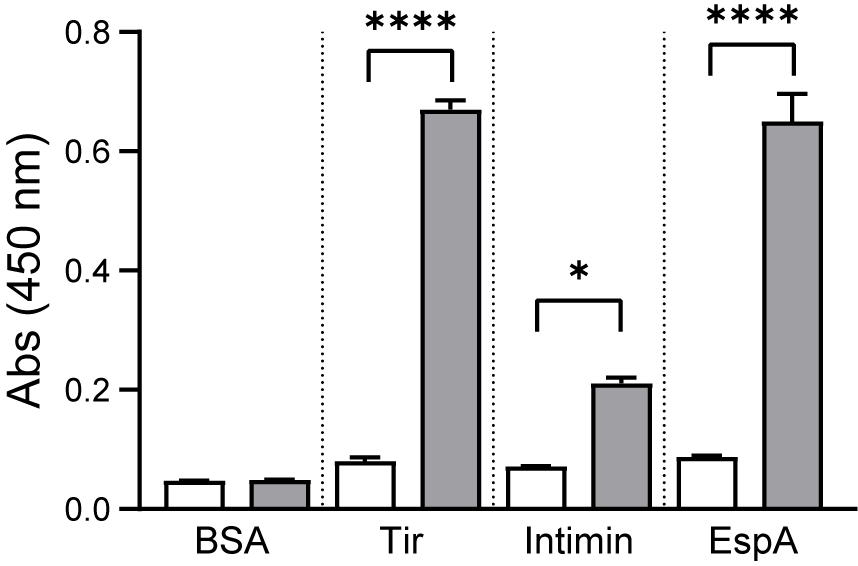

Supplement: S6 Fig — Reactivity of IgG antibodies was tested by coating ELISA plates with individual purified antigens, in addition to BSA as negative control. Sera from pre-immune and one-dose EIT-PF immunized steers (week 2 p.i.) were pooled and assayed in triplicate, and the averages (±SEM) are shown. Data were analyzed by One-way ANOVA and Sidak’s post hoc contrasts; asterisks indicate a p-value < 0.05 (*) or < 0.0001 (****). (TIF) [file pone.0341075.s006.tif]
